# Use of a pLDH-based dipstick in the diagnostic and therapeutic follow-up of malaria patients in Mali

**DOI:** 10.1186/1475-2875-10-345

**Published:** 2011-11-24

**Authors:** Amed Ouattara, Safiatou Doumbo, Renion Saye, Abdoul H Beavogui, Boubacar Traoré, Abdoulaye Djimdé, Amadou Niangaly, Kassoum Kayentao, Mouctar Diallo, Ogobara K Doumbo, Mahamadou A Thera

**Affiliations:** 1 Malaria Research and Training Center, Department of Epidemiology of Parasitic Diseases, Faculty of Medicine, Pharmacy and Dentistry, University of Bamako, Mali

**Keywords:** malaria, diagnostic, drug efficacy follow-up

## Abstract

**Background:**

Malaria is a major public health problem in Mali and diagnosis is typically based on microscopy. Microscopy requires a well trained technician, a reliable power source, a functioning microscope and adequate supplies. The scarcity of resources of community health centres (CHC) does not allow for such a significant investment in only one aspect of malaria control. In this context, Rapid Diagnostic Tests (RDTs) may improve case management particularly in remote areas.

**Methods:**

This multicentre study included 725 patients simultaneously screened with OptiMal-IT test and thick smears for malaria parasite detection. While evaluating the therapeutic efficacy of choroquine in 2 study sites, we compared the diagnostic values of thick smear microscopy to OptiMal-IT test applying the WHO 14 days follow-up scheme using samples collected from 344 patients.

**Results:**

The sensitivity and the specificity of OptiMal-IT compared to thick smear was 97.2% and 95.4%, whereas the positive and negative predictive values were 96.7 and 96.1%, respectively. The percent agreement between the two diagnostic tests was 0.93. The two tests were comparable in detecting malaria at day 0, day 3 and day 14. The only difference was observed at day 7 due to high gametocytemia. Subjectively, health care providers found OptiMal-IT easier to use and store under field conditions.

**Conclusion:**

OptiMal-IT test revealed similar results when compared to microscopy which is considered the gold standard for malaria diagnostics. The test was found to have a short processing time and was easier to use. These advantages may improve malaria case management by providing a diagnostic and drug efficacy follow-up tool to peripheral health centres with limited resources.

## Background

In sub-Saharan Africa, malaria is responsible of 25% of all cause mortality in children less than 5 years old [1]. Studies conducted by the Department of Epidemiology of Parasitic Diseases at the University of Bamako, Mali in collaboration with the National Malaria Control Programme (NMCP) of Mali, have demonstrated that the prevalence of malaria in rural areas was more than 70% in children under five years of age and the incidence of clinical infection ranges from 1.5 to 2 episodes per child per year [2]. In addition, severe malaria was reported to account for 15% of hospitalizations in children between the ages of 0 and 14 years in the capital city of Bamako leading to a case fatality rate of approximately 17% at the National Paediatric Hospital in Bamako, and 25% countrywide [3,4].

Mali health policy makers have prioritized improved access to accurate diagnosis and early treatment of malaria with the aim to reduce disease burden and lower the high case fatality rate. Challenges to this strategy are many and include the absence of diagnosis or delay in diagnosis, which may lead to an evolution of simple malaria cases into more severe forms. In Mali, malaria diagnosis is mostly based on microscopy, which requires a power source, a microscope, staining solution, and a well trained technician. To maintain the capacity to provide precise and reproducible thick smear results to the population a large initial investment is required followed by regular assessment and frequent training of microscopists throughout this largely rural country. With the scarce resources of community health centres (CHC), reliable, accurate and accessible microscopy capacity is rare at the community level. Health workers and CHC budgets must also ensure that other malaria control strategies such as insecticide-impregnated bed nets are also available to their target population. Moreover, slide staining and reading times are relatively long when compared to RDTs processing time. This delay in diagnoses may also delay treatment initiation, thus worsening the prognosis of infected children. The absence of malaria diagnostics may also lead to an over-diagnostic and treatment that jeopardizes the effectiveness of available anti-malaria drugs.

During the last decade, rapid diagnostic tests (RDTs) using chromatography and ELISA technology have been developed and largely used in field studies [5-7]. These diagnostic strips are based on antigens expressed by the parasite during the erythrocyte stage such as histidine-rich protein-2 (HRP2) [8-10] or lactate dehydrogenase (LDH) [11]. HRP2 is involved in haemozoin formation, while [12] Plasmodium falciparum LDH catalyzes the conversion of lactate into pyruvate and nicotinamide adenine dinucleotide (NADH) [13], a reaction essential to parasite survival. LDH is produced only by living parasites [14] as they need energy to ensure their development in the course of the asexual life cycle. The enzyme is short lived with a half-life of about 2 to 4 days. Some advantages of LDH-based RDT compared to HRP2-based tests are detection of current infections and discrimination of P. falciparum species from non-falciparum species that cause less severe disease.

The purpose of this study was to evaluate the utility of parasite-based LDH (pLDH) RDT in Mali under field conditions, to calculate the diagnostic values (positive and negative predictive value and, sensitivity and specificity) of the RDT compared to microscopy and to assess the accuracy of the RDT as a diagnostic method in malaria drug efficacy studies using the WHO 14 days *In vivo *protocol. This study was conducted in 2003 when choroquine was still the first line malaria treatment in Mali.

## Methods

### Study areas and period

Four health centres participated to this multicentre study; the community health centre of Faladie, a village located 80 km north-west of Bamako; the health clinic of Kolle, a village situated 60 km south-west of Bamako; the paediatric intensive care unit of Gabriel Touré Hospital in Bamako; Point G hospital (PGH) and the secondary health centre of District V in Bamako. The study was conducted simultaneously in all health centres during a period of intense malaria transmission from September 2003 to December 2003.

### Study population and design

A total of 725 patients were enrolled in the study with a sex ratio of 1.13:1 female to male. The study population in Faladie consisted of children aged 0 to 9 years attending the health centre for malaria related symptoms and asymptomatic pregnant women attending the clinic for prenatal visits. In Kolle, all patients attending the clinic were included in the study without any age restriction. The study population in Gabriel Touré Hospital consisted of children admitted to the paediatric ward with fever. Patients from Point G hospital (PGH) were referred to the Department of Epidemiology of Parasitic Diseases Laboratory due to the proximity of the two institutions. At the health centre of District V, the study population included pregnant women attending the prenatal clinic and a scheduled vaccination programme.

After administering the informed consent, participants were allocated an identification number and clinically examined by the study doctors. In all these sites, participants received a finger prick in order to make a thick smear and the RDT. In Faladie and Kolle, patients positive for malaria by thick smear or RDT were given choroquine and asked to attend the clinic at days 1, 2 3, 7 and 14 for efficacy assessment. The study was approved by the ethical committee of the Faculty of Medicine, Pharmacy and Dentistry of Bamako, Mali.

### Laboratory techniques

A thick smear was made for each patient and stained with 3% Giemsa for 45 minutes. Parasite density was obtained by counting the number of parasites per 300 leukocytes and using an estimate of 7500 leucocytes per mm^3 ^of blood for quantification. OptiMal-IT was used according to the procedure recommended by the manufacturer.

### Drug efficacy follow-up

In Kolle and Faladie, participants were followed for 14 days for drug efficacy assessment. After the initial testing and drug administration, patients were asked to return on days 3, 7 and 14. All the 3 doses of the choroquine were given at the health centre. Sulfadoxine-pyrimethamine (SP) was used as second-line treatment in a single dose of 1.25mg/kg when either the thick smear and/or OptiMal-IT was positive during patient follow-up. Efficacy outcomes were assessed according to the WHO definitions [15]; early therapeutic failure (ETF), late therapeutic failure (LTF) and satisfactory clinical response (SCR). The three levels of parasitological response as defined by WHO [15] were also used.

### Quality control and OptiMal-IT acceptability

All thick smears were stained and read immediately at the study site by trained microscopists. Smears were then read by a second microscopist for quality control. Additionally, 10% of thick smears were re-examined by a third reader. OptiMal-IT tests were read independently by two technicians. A third reader was used as a tie breaker if the two readers disagree on their reading. To assess the providers experience with the RDT, health care providers were asked to complete a brief questionnaire which aimed to collect their feedback on the test and its ease of use. The questionnaire was self-reported, anonymous and conducted after the test completion.

### Data analysis

Data were recorded on case report forms and laboratory logs sheets. Data were analyzed using Access 2007 and SPSS 13.0 software. We used a Chi-Squared or Fisher's exact test to compare frequencies of malaria detected cases. Kappa statistics were used to assess test concordance. The sensitivity, specificity and the predictive values of both tests were also computed using microscopy as the gold standard.

To assess the relationship between parasite density and OptiMal-IT results, we defined 4 groups of parasitemia (25-1,000 parasites/mm^3^; 1,025-2,500 parasites/mm^3^; 2,525-5,000 parasites/mm^3 ^and more than 5,000 parasites/mm^3^). The correlation between OptiMal-IT detection level and parasites count was assessed for each of these groups.

### Ethical considerations

Ethical approval was granted by the Faculty of Medicine, Pharmacy and Dentistry IRB Committee of the University of Bamako, to carry out this research. Written informed consent was obtained from all individuals participating in interviews. Confidentiality was maintained with use of study number during the data analysis process.

## Results

**Figure 1:**
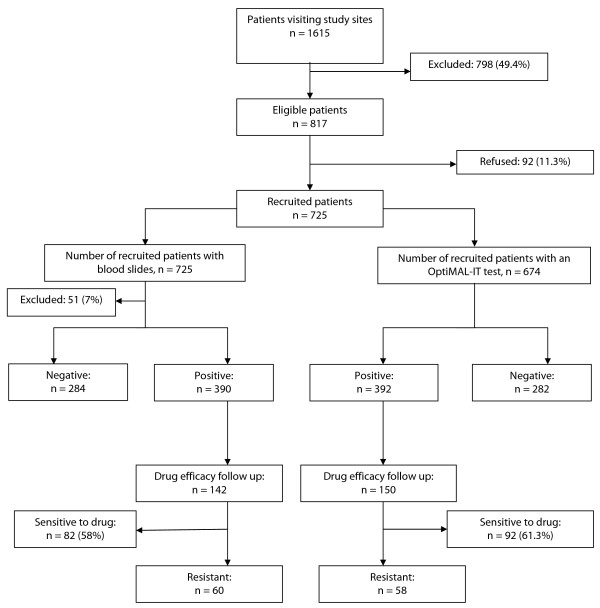
**Study profile of study patients visit at study site**. OptiMal-IT, thick smear test and drug efficacy follow-up outcome.

The distribution of participants by study site is shown in Table 1. Gabriel Toure Hospital (GTH) and Point G hospital (PGH) population were comprised of children less than 10 years old, while in District V, the health clinic centre offers services only to pregnant women. Antimalarial drug efficacy follow-up was conducted only in Faladie and Kolle (142 patients included out of 725 (19.6%). Study participants attendance and follow-up are described in Figure 1.

**Table 1:** Distribution of study patients by study site and sex.

Study site	Female	Male	Total
Faladie	111	154	265
Gabriel Toure Hospital	53	73	126
Point G Hospital	68	84	152
Kolle	51	29	80
District 5 medical centre	102	0	102
Total	385	340	725

### Parasite detection and level of resistance

Overall, our results indicated that 56.5% of patients presenting with fever or other malaria clinical symptoms had a positive thick smear, whereas 58.2% were positive by OptiMal-IT. Table 2 summarizes the comparison between thick smear microscopy and OptiMal-IT test results in all study sites.

**Table 2:** Distribution of thick smear and OptiMal-IT malaria positive cases by study site

Study site	Thick smear n (%)	OptiMal-IT n (%)	p value
Faladie	266 (78.6)	263 (77.6)	0.78
Gabriel Toure Hospital	104 (64.4)	104 (74)	0.13
Point G Hospital	151 (26.5)	137 (30.7)	0.43
Kolle	78 (84.6)	75 (81.3)	0.58
District 5 medical centre	97 (11.3)	100 (11.9)	0.90

Based on the WHO classification, 58% of patients have parasites sensitive to choroquine and patients with early treatment failure (ETF) were observed in 6% of cases. Using the same clinical resistance definition, late treatment failure (LTF) was reported in 12% of participants while late parasitological failure (LPF) was reported in 24% of participants (table 3).

**Table 3:** Levels of parasitological and clinical resistance determined by thick smear

Level of resistance				
	**ETF (%)**	**LTF (%)**	**LPF (%)**	**ACPR (%)**

Thick smear	9 (6)	17 (12)	34 (23)	82 (58)

ETF; Early treatment failure, LTF; Late treatment failure, LPF; Late parasitological failure, ACPR; Adequate clinical and parasitological response.

Among the 102 pregnant women who attend the District V health clinic, 12 malaria cases were diagnosed using thick smear, while OptiMal-IT test revealed eleven cases of malaria.

### Diagnostic parameters

OptiMal-IT identified 13 malaria cases which were not diagnosed by microscopy, whereas thick smear analyses revealed 11 samples which were negative by OptiMal-IT. Overall, using thick smear as the reference technique the sensitivity of OptiMal-IT was 97.2% (95% CI, 0.96-0.99), with a specificity of 95.4% (95% CI, 0.93-0.98). Positive and negative predictive values were 96.7% (95% CI, 0.95-0.98) and 96.1% (95% CI, 0.94-0.98) respectively. Concordance (κ) was 0.93 (table 4).

**Table 4:** Diagnostic values of OptiMal-IT compared to thick smear as reference technique.

Thick smear							
	**Positive**	**Negative**	**Sensitivity (%)**	**Specificity (%)**	*PPV (%)	**^+^NPV (%)**	**Frequency of positive (%)**

OptiMal-IT positive	379	13	97.2	95.4	96.7	96.1	58.2
OptiMal-IT negative	11	271					
Frequency of positive (%)	56.5						

*PPP; Positive predictive value; NPV; ^+^Negative predictive value

### OptiMal-IT detection levels

OptiMal-IT was able to identify 97.2% of malaria cases detected by thick smear at all parasite densities. All malaria cases with parasitemia higher than 1000 parasites/μl were detected by OptiMal-IT test (table 5). In addition to asexual stage, *P. falciparum *gametocytes were also diagnosed by the rapid test. Importantly, species other than *P. falciparum *were identified. *Plasmodium ovale *(*P. ovale*) infections were only detected at 2525 parasites/μl and above. *Plasmodium malariae *(*P. malariae*) infections were fully detected at 2500 parasites/μl and beyond with only 40% of infections below this treshold detected.

**Table 5:** Relationship between *Plasmodium falciparum *parasites density and OptiMal-IT

Parasitemia per mm3 of blood				
	**25-1000** **n (%)**	**1025-2500** **n (%)**	**2525-5000** **n (%)**	**>5000** **n (%)**

OptiMal-IT positive	41 (89.1)	27 (100)	22 (100)	259 (100)
OptiMal-IT negative	5 (15.9)	0 (0)	0 (0)	0 (0)

### Dynamics of pLDH after malaria treatment

**Figure 2:**
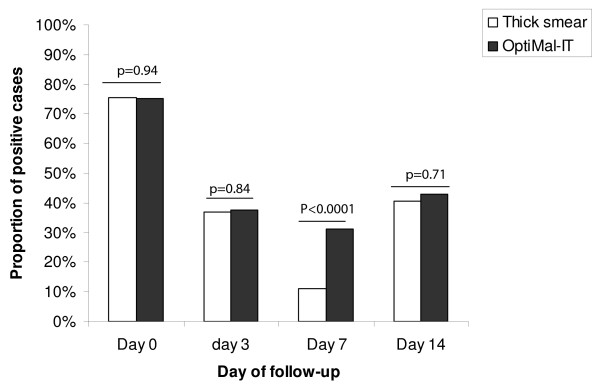
**Dynamics of OptiMal-IT test positivity during patient follow-up after a malaria treatment**. The day of follow-up is on the × axis and the proportion of positive cases on the y axis. Results from both tests were comparable during follow-up except for day 7 when the proportion of cases detected by OptiMal-IT was statistically higher compared to the proportion observed with thick smear

There was no significant difference between diagnostic techniques at day 0 (odd ratio: 0.99, 95% CI: 0.70-1.40), day 3 (odd ratio: 1.05, 95% CI: 0.65-1.70) and day 14 (odd ratio: 1.10, 95% CI: 0.68-1.80) respectively (Figure 2). OptiMal-IT had a higher false positive test 7 days after the treatment compared to thick smear (odd ratio: 3.70, 95% CI: 1.94-7.04) (Figure 2).

### Acceptability of the test by health providers

Health providers had previous knowledge and some experience with the OptiMal-IT test and found it easy to use and store. In fact, more than 99% were able to use the test after a training of one hour compared to at least a week when laboratory technicians are trained to discriminate malaria species using microscopy. OptiMal-IT storage at room temperature was described to be an advantage (table 6).

**Table 6:** OptiMal-IT test approval by health care providers

	Perception of test quality	Number of respondents	Frequency
Processing	Easy	718	99.3
	Acceptable	5	0.7
	Difficult	0	0
Handling and storage	Good	708	99.2
	Acceptable	5	0.7
	Poor	1	0.1
Prior knowledge about the test	Yes	528	77.8
	No	151	22.2

## Discussion

Our Findings suggest that OptiMal-IT test is an effective tool in the diagnosis of malaria. We also found that the test was effective in the follow-up of malaria drug efficacy with some limitations due to the discrepancy observed at day 7. Finnaly, we found that health care providers have a good understanding about the test and field practicians sites preferred OptiMal-IT test compared to thick smear.

We have found that OptiMal-IT was comparable to thick smear microscopy which remains the current gold standard for malaria diagnosis. In an earlier study of the first generation OptiMal-IT test in western Uganda, Jelinek et al [16] observed a test specificity of 62.2% and a sensitivity of 58.8%, well below what was observed in this study. This difference may be explained by the difference in the accuracy of the thick smear reading and the improvements made in the design of the second generation OptiMal-IT test [17]. Palmer et al [18] and Dolo et al [19] observed results similar to our findings in field studies conducted respectively in Honduras and in Mali with analogous sensitivity, specificity and Kappa concordance. Our thick smears were read by 2 independent readers with one at the field site and a second more experienced reader in the laboratory in Bamako. On discordant cases, a third reader defined as a tie breaker was asked to confirm or infirm the results. The same reading method was used to determine OptiMal-IT test positivity. This quality control may have improved our data by removing false positive thick smears and OptiMal-IT tests, thus improving our positive and negative predictive values. This high positive predictive value is reflective of the high prevalence of malaria during malaria transmission season.

OptiMal-IT test was found sensitive and specific for malaria infection in a variety of populations in Mali. Similar observations were made by Maltha *et al *[20] when OptiMal-IT test was compared to other RDTs. This would support the use of OptiMal-IT test to detect malaria in health centres without access to microscopes. Recent studies conducted by WHO [21] and research teams in Africa [22,23] concur with our findings.

When we assessed the ability of OptiMal-IT to detect malaria during pregnancy, we found that eleven out of the 12 cases of malaria in this group of 102 pregnant women detected by thick smear were diagnosed by OptiMal-IT. This reveals the great advantage OptiMal-IT test may have in women at risk of having adverse fetal outcomes due to malaria in pregnancy. Findings by Limanggeni et al [24] suggest that a positive maternal OptiMal-IT test is a predictor of low birth weight in Malawi newborns. These observations are supportive of the used of OptiMal-IT test as an adequate screening tool for the detection of clinically significant parasitemia in the pregnant population.

Similarly to a study conducted by Cooke et al [25], we found that OptiMal-IT test sensitivity increases with parasitemia. All *P. falciparum *malaria cases with parasitemia greater than 1000 trophozoites/μl of blood were detected as previously described [20]. However, almost 16% of blood stage asexual forms with parasitemia less than 1000/μl were not detected. This observation highlights the fact that an improvement needs to be done to reduce the detection limit of this RDT. Additional improvement may include the quality of packaging and the accuracy of information in the kit [26]. If these improvement are accomplished it may allowed the use of this test as a screening tool as proposed by Ishengoma *et al *[27]. Moody et al [7] reported that one of the pan-specific monoclonal antibodies has a lower affinity in attaching to *P. ovale *antigens. This hypothesis is supported by the genetic diversity of the encoding gene which may prevent the binding of monoclonal antibodies to their targets [28].

The rapid clearance of pLDH enzyme following successful treatment was documented in participants who enrolled in a 28 days drug efficacy study [29]. We observed a significant difference between thick smer and OptiMal-IT in the frequency of a positive test at day 7 after treatment. This difference can be explained by the exclusive presence of *P falciparum *gametocytes in twelve participants samples which were not taken into account in trophozoites counting. Detection of gametocytes is the major drawback of OptiMal-IT in drug efficacy monitoring [30]. Previous studies [29,31] have found similar results prompting the question whether this observation may limit the use of pLDH based tests in the monitoring of drug efficacy. However, malaria is known to be lethal in children if not detected and treated promptly. Most cases occur in areas where there is limited access to health clinics and almost no access to microscopes and power sources. With the introduction of ACTs, pLDH-based RDTs may provide tools for malaria detection as well as treatment efficacy in these remote areas as recommended my Houze *et al *[29]. Results confirmation may be provided by regional reference centres which have tools to diagnose and quantify parasites density. With the increased use of ACTs, we expect less false-positive RDT results due to gametocytes because artemisinins are gametocytocidal and gametocytemia on day 7 is rare [32].

Finally, we found that clinicians in remote clinics as well as in reference hospitals are familiar with RDTs. Acceptance of RDT by health care providers and the need to limit antimalarial drug administration to only infected patients in a timely manner supports the implementation of pLDH-based RDTs in rural clinics and health centres without microscopy.

## Conclusion

Even though its limitations for malaria drugs efficacy follow-up related to its inability to count parasite load and the positivity of the test in presence of gametocytes, OptiMal-IT has a good Kappa concordance. The test may be suitable for therapeutic follow-up of patients in areas without access to microscopes or a power source to use conventional malaria diagnostic techniques. However, a confirmation of clinical resistance levels of these remote areas using thick smears results should be required. In addition, the ability of the test to detect parasites at the gametocytes stage may be a great advantage when patients will be screened during low transmission season in order to identify gametocytes carriers. These carriers may be treated with adequate drug to reduce transmission during the raining season.

## Abbreviations

CHC: community health centre; RDT: rapid diagnostic test; WHO: World health organization; NMCP: National malaria control program; pLDH: plasmodium lactate dehydrogenase; HRP2: histidine rich protein-2; ELISA: Enzyme-linked immunosorbent assay; HGT: hospital Gabriel Touré.

## Competing interests

The authors declare that they have no competing interests.

## Authors' contributions

OKD designed the study, coordinated study execution, scientifically reviewed the paper and approved the final draft. OA reviewed the design of the study, supervised coordination of the study, wrote the report drafts and approved the final version. DM reviewed the design of the study, supervised coordination of the study, reviewed the report drafts and approved the final version. SD, RS, AHB and AN conducted the field study, reviewed the report drafts and approved the final version. KK did statistical analysis, scientifically reviewed the paper and approved the final draft. BT, MAT and AD designed the study, scientifically reviewed the paper and approved the final draft.
